# Training climate matters: programmatic openness, individual belonging, and trainee mistreatment in graduate medical education

**DOI:** 10.1080/10872981.2026.2687300

**Published:** 2026-06-12

**Authors:** TingLan Ma, Binbin Zheng, Michael Soh, Jerri Curtis, Steven J. Durning, Ting Dong

**Affiliations:** a Department of Health Professions Education at the Uniformed Services University of the Health Sciences in Bethesda, Bethesda, MD, USA; b Health Professions Education Program, School of Graduate Studies, University of Maryland at Baltimore, Baltimore, MD, USA; c National Capital Consortium, Uniformed Services University of the Health Sciences in Bethesda, Bethesda, MD, USA

**Keywords:** Wellbeing, burnout, mistreatment, programmatic openness, belonging

## Abstract

**Background:**

Medical trainee mistreatment remains a widespread challenge in graduate medical education, contributing to burnout, depression, and workforce attrition. Graduate medical education programs have sought to create safer learning environments by fostering programmatic openness—climates perceived as transparent, welcoming, and supportive. Yet it remains unclear how such programmatic conditions interact with individual experiences of belonging to shape wellbeing. Drawing on ecological systems theory, we examine how programmatic openness and trainee belonging interact to mitigate trainee mistreatment during residency.

**Methods:**

We conducted an anonymous online survey (Dec 2023–Jan 2024) at three large US teaching sites with 62 GME programs. Scales with robust validity evidence measured perceived programmatic openness, individual belonging, perceived mistreatment, burnout, and social support. We built a latent moderation model, encompassing both confirmatory factor analysis and latent interaction effects. We examined how programmatic openness moderated trainee perceived mistreatment while controlling for social support and trainee demographics.

**Results:**

Of 675 eligible trainees, 266 responded (39.4%). Measurement models demonstrated good fit, supporting construct validity. Both programmatic openness and belonging were protective against perceived mistreatment, which was strongly associated with burnout. Residents’ belonging to the training program was significantly associated with low burnout, even after accounting for social support. Moderation analyses revealed that programmatic openness most strongly buffered mistreatment among trainees who experienced low belonging, with diminished effects at higher belonging levels.

**Conclusion:**

Findings revealed a compensatory effect, suggesting the beneficial effects of programmatic openness were especially crucial among trainees who experienced low individual belonging. This finding aligns with the ecological system theory, which posits that supportive environments may mitigate individual-level vulnerabilities. Both burnout and perceived mistreatment were at its lowest when programmatic openness and individual belonging were both present. This underscores the need for residency programs to foster both openness and belonging as core strategies to support trainee wellbeing.

Medical trainee mistreatment is a prevalent and harmful issue worldwide in graduate medical education (GME) [1–5], cutting across specialties and training programmes [6–9]. Encompassing behaviours like bullying, exclusion, and humiliation, mistreatment is associated with adverse outcomes such as burnout, depression, increased medical errors, and suicidal ideation [10–13]. Among these, burnout—characterised by emotional exhaustion and disengagement—is linked to workforce attrition and compromised patient care [14,15].

Following a 2021 call from the Accreditation Council for Graduate Medical Education (ACGME) [16] to lower discrimination and increases community building, many GME programmes have sought to promote safer learning environments [17–20]. Central to these efforts is programmatic openness, which we define as the extent to which training programmes cultivate transparent, welcoming, and psychologically safe climates [21]. Programmes embracing openness aim to create environments where trainees feel safe to express concerns, seek help, and bring their authentic identities into their professional development without fear of marginalisation. While openness is a programme-level construct, we operationalise it through trainees’ reported perceptions of their programme's commitment. This approach recognises that, while commitment and lived climate are distinct, trainee perception is a meaningful and measurable indicator of institutional context.

Approaches to fostering programmatic openness may vary. Some programmes emphasise interpersonal strategies to build trust and create open dialogue, while others focus on overhauling systemic issues, including revising institutional policies and making explicit commitments to embracement and belonging [17,19,20]. Despite these varying initiatives, it remains underexplored whether programmatic openness—as reflected in trainees’ perceptions of their programme’s *commitment and efforts* to fostering an open and inclusive climate—are associated with trainee wellbeing.

While programme-level efforts signal institutional commitment, they do not always translate directly to individual-level experiences [22]. Ecological systems theory [23,24] highlights how individual outcomes are shaped by interactions between macro-level factors (e.g., programme culture) and micro-level experiences (e.g., individual belonging). Programmatic openness represents the macro-level institutional climate, whereas a trainee’s sense of belonging [25]—defined as an individual experience of feeling accepted, valued, and connected within the community—serves as the micro-level manifestation of that environment [25,26]. Prior research has shown that micro-level factors, including social support [27–29], and belonging [26] are protective in high-stress environments. However, although a growing body of medical education literature has examined the educational environment and learning climate [17,19,20,30–32], less is known in GME about how programme-level conditions interact with individual experiences to shape trainee wellbeing.

We propose that programmatic openness and trainees’ sense of belonging interact to shape experiences of mistreatment. The presence of both openness and belonging is likely to produce an additive effect, creating a more psychologically safe and supportive environment for trainees. Conversely, if one protective factor is absent, the other may offer a compensatory effect, with high programmatic openness buffering against low belonging, and vice versa [33,34]. This ecological dynamic between institutional climate and individual belonging has received limited empirical attention in GME and warrants further investigation.

Without this evidence, GME programmes may risk investing in micro-level wellness strategies while overlooking the specific role that macro-level initiatives could play in mitigating mistreatment and burnout. This may limit the effectiveness of efforts to support trainee wellbeing. To address this gap, our study explores the following research question: how are programme-level openness and individual-level belonging related to perceived mistreatment and burnout? Specifically, how does the interplay between programmatic openness and individual belonging relate to trainee mistreatment in GME? [23,24][33,34]. We consider lower reported mistreatment and burnout as key indicators of wellbeing, recognising that they are not the only determinants of trainee health.

## Methods

### Participants and study context

We conducted an anonymous online survey across three affiliated GME institutions in the northeastern United States. Linked through a shared training network, these sites hosted more than 62 GME programmes and approximately 675 trainees, including interns, residents, and fellows. Programmes spanned diverse clinical specialties, including primary care specialties (e.g., Internal Medicine, Family Medicine), surgical and procedural specialties (e.g., General Surgery, Anaesthesiology), and hospital-based diagnostic specialties (e.g., Radiology, Pathology). The three sites represented distinct training environments: a large tertiary academic medical centre, a regional community-based hospital, and a health sciences university setting. All trainees affiliated with these institutions were invited to participate. While specialty-specific workflows and day-to-day clinical demands likely varied across these sites, the programmes were situated within a shared training structure and a broadly similar regional sociopolitical context.

We examined how trainees’ perceived programmatic openness and sense of belonging related their experiences of perceived mistreatment. Burnout was included as a co-occurring outcome, and social support was entered as a control variable to better isolate the unique contribution of programmatic openness. The study was reviewed and determined to be exempt by the Uniformed Services University Institutional Review Board (IRB protocol DBS.2023.596).

### Recruitment

Because the study involved an anonymous online survey and was deemed exempt, written informed consent was not required. Instead, implied consent was used: participants were provided with a study information page describing the purpose of the study, the voluntary nature of participation, and safeguarding plans for confidentiality, and consent was implied by participants’ decision to proceed with the survey. Data collection took place between December 2023 and January 2024, with an anonymous Qualtrics survey link distributed via the email distribution lists of interns, residents, and fellows, with two reminder emails sent at two-week intervals after the initial invitation. No incentives were provided for participation.

### Measures

The survey included validated existing measures (detailed below) and demographic items, such as sex, training year, and specialty. To refine the flow, relevance, and language appropriateness, the team recruited four medical and surgical trainees from the institution to pilot the survey and conduct cognitive interviews. Based on feedback, adjustments were made, including clarified item wording and additional demographic response options. Participants were explicitly instructed to skip any questions they did not wish to answer to uphold voluntary participation. Key measures are detailed below:

**Mistreatment**. Trainee mistreatment was assessed using the 6-item Negative Acts Questionnaire-Revised (NAQ-R) scale [35]. The NAQ-R measures workplace bullying, defined as ‘repeated actions and practices directed against one or more workers, which are unwanted by the victim, carried out deliberately or unconsciously, and result in humiliation, offence, distress, or interference with work performance, creating an unpleasant working environment.’ This scale has been previously utilised in national assessments of workplace bullying, including studies involving US surgical residents [36], and the Cronbach’s *α* with the current sample is .821.

**Programmatic openness.** Programmatic openness was assessed using a 7-item scale [21] that measured the extent to which a trainee perceived their GME programme’s and commitment and efforts in fostering an open and accepting environment. We modified the original instructions by replacing references to ‘organisation’ with participants’ ‘training programme’ (e.g., *My training programme* ‘*tries to create an awareness and appreciation of individual and cultural differences among all residents*’). The measure was assessed using a five-point Likert scale (1 = *strongly disagree* to 5 = *strongly agree*) and demonstrated high internal consistency (Cronbach’s *α* = .895).

**Individual Belonging.** Trainee perception of individual belonging were assessed using a 4-item scale [26] (e.g., ‘*I feel comfortable voicing ideas and opinions*’, ‘*I feel valued and encouraged to be myself by people around my work unit*’). The measures used a five-point Likert scale (1 = *strongly disagree* to 5 = *strongly agree*) and demonstrated high internal consistency (αs = .880).

**Burnout.** To assess residents’ feelings of wellbeing and burnout, we used the 7-item Medical Student Well-being Index (MSWBI) [37], modifying the wording by replacing ‘medical school’ with ‘training programme’ (e.g., *Do you feel burned out from your training programme?*). The response format was expanded from a binary (Yes/No) to a 5-point Likert to capture a broader range of perceptions and intensities. This scale demonstrated good internal consistency with current sample (*α* = .865).

**Social Support.** Social support was assessed using 4-item Brief Social Support Scale [38]. (e.g., ‘*how often is someone available to allow you to vent your emotion in front of this person?*’). Responses were recorded on a 4-point Likert (1 = *never* to 4 = *always available*) with good reliability (*α* = .968).

### Data analysis

**Figure 1.**
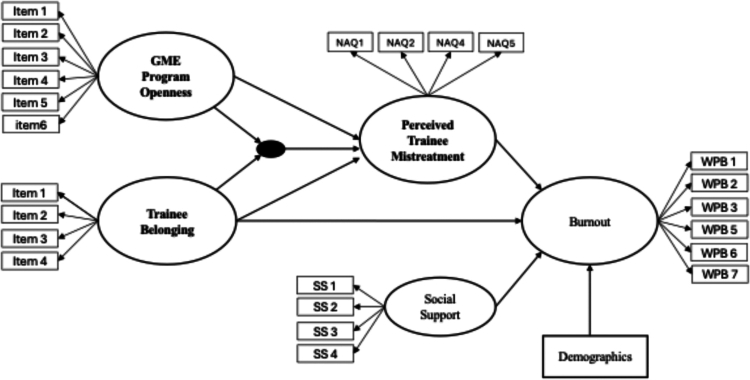
Generic model of latent moderation structural equation (LMS).

We used Mplus 8.0 to build the statistical model in two steps. First, we estimated a measurement model of key constructs (Figure 1), including latent variables of mistreatment, burnout, programmatic openness, belonging, and social support. Estimation of latent variables accounted for both observed indicators and measurement error, thereby providing more unbiased estimates. We evaluated model fit using Hu and Bentler's [39] criteria, including chi-square, comparative fit index (CFI), root means square residual error of approximation (RMSEA), and Standardised Root Mean Square (SRMR). Good fit was indicated as CFI ≥ 0.95, RMSEA ≤ 0.06, and SRMR ≤ 0.08 [39].

Second, we specified a latent interaction model with the latent moderated structural equations (LMS) method [40,41], adding structural paths to assess effects of programmatic openness, belonging, and their interaction as predictors. Trainee mistreatment was modelled as the primary outcome of programmatic openness, with burnout as a co-occurred, distal outcome, controlling for social support and demographics. We built this model based on empirical evidence [14,15,24,28] and theories that guided our hypotheses [33,42]. Full Information Maximum Likelihood (FIML) estimation was used to handle missing data. Individual characteristics (e.g., sex, training years) were included as auxiliary variables to help estimate missingness.

Because latent interaction models within the LMS framework [40,43] do not yield conventional SEM fit indices (e.g., CFI, RMSEA), the model with measurement and structural paths served as the baseline. After establishing a well-fitted baseline model, we estimated moderation by examining the interaction term (Figure 1). For significant interaction effects, simple slopes were probed and plotted using conventional values recommended by Aiken and West: [44] 1 *SD* below the mean, the mean, and 1 *SD* above the mean. These values were used to aid interpretation and visualisation of the interaction and were not treated as categorical cutoffs for belonging or programmatic openness.

## Results

We received 266 responses from 675 medical trainees (response rate = 39.4%). As shown in Table 1, the demographic profile of respondents was comparable to the sites’ overall population. The sample was predominantly male (67.5%) and White (73.4%). Among respondents, 9.7% identified as Asian, 5.8% as multiracial, 5.3% as Hispanic or Latino/a/x of any race, 5.3% as Black or African American, and 0.5% as American Indian or Alaska Native. Junior trainees (Year 1 and 2) comprised 42.2% of the sample, while 57.8% were senior trainees (Year 3 and above). The most frequently reported specialties were Family Medicine (*n* = 23, 11.4%), Internal Medicine (*n* = 23, 11.4%), Anaesthesiology (*n* = 19, 9.5%), General Surgery (*n* = 16, 8.0%), and Psychiatry (*n* = 13, 6.5%). Response counts varied across items since all survey questions were optional. We conducted Little’s Missing Completely at Random (MCAR) test and missing completely at random assumption was met, χ^2^(729) = 658; *p* = .97, qualifying the use of FIML to account for missing data.

**Table 1.** Participant characteristics.

Participant characteristics	*N* (%)
**Gender**	
Female	68 (32.1%)
Male	143 (67.5%)
Transgender	1 (0.5%)
**Race/Ethnicity**	
Hispanic or Latino or Spanish origin of any race	11 (5.3%)
American Indian or Alaskan Native	1 (0.5%)
Asian	20 (9.7%)
Black or African American	11 (5.3%)
White	152 (73.4%)
Two or more races	12 (5.8%)
**Training years**	
Residency Year 1 and 2 (Junior trainees)	86 (42.2%)
Residency Year 3 and above (Senior trainees)	118 (57.8%)
**Specialty**	
Surgical specialties	19 (8.4%)
Non-surgical specialties	206 (91.6%)

After specifying the full measurement model, the model fit the data moderately, χ^2^(338) = 656.054, *p* < .001, CFI = .905, RMSEA = .06 [90% CI = .058 to .073], SRMR = .079. Factor loadings were examined (Table 2) and loadings below .45 were excluded (Bentler’s rule, see Table 2 notes). Model modification indices were examined to allow inter-scale items to be correlated when it’s appropriate and theoretically justifiable. For examples, burnout item 3 (*feeling depressed or hopeless*) and item 6 (*feeling anxious or irritable*) were allowed to be correlated within the model to improve fit. Similarly, programmatic openness item 5, reflecting awareness and appreciation of individual differences, and item 6, reflecting understanding feelings about people who are different, were also allowed to be correlated. No cross-scale items were allowed to be correlated. The final adjusted model yielded improved fit, Δχ² = −254.574, Δdf = −81, ΔCFI = .050, ΔRMSEA = −.009, and ΔSRMR = −.012, and demonstrated an overall good fit to the data, χ^2^(257) = 401.480, *p* < .001, CFI = .955, RMSEA = .051 [90% CI = .041 to .06], SRMR = .067, indicating that this final confirmatory factor structure was adequate and consistent with the theoretical and empirical foundations of the literature.

**Table 2.** Unstandardised coefficients of latent moderation model.

Factor loadings	Estimate	S.E.	*P*-value	R-square
**Perceived Mistreatment**				
NAQ 1	1	0	999	0.787
NAQ 2	0.737	0.057	*p* < .001	0.652
NAQ 4	0.997	0.099	*p* < .001	0.441
NAQ 5	0.568	0.049	*p* < .001	0.538
**Burnout**				
WPB 1	1	0	999	0.627
WPB 2	0.943	0.078	*p* < .001	0.5
WPB 3	1.18	0.111	*p* < .001	0.65
WPB 5	0.986	0.098	*p* < .001	0.539
WPB 6	0.987	0.113	*p* < .001	0.454
WPB 7	0.893	0.099	*p* < .001	0.441
**GME Programmatic Openness**				
Item 1	1	0	999	0.811
Item 2	0.972	0.053	*p* < .001	0.761
Item 3	0.722	0.06	*p* < .001	0.613
Item 4	0.941	0.065	*p* < .001	0.721
Item 5	0.973	0.064	*p* < .001	0.769
Item 6	0.942	0.07	*p* < .001	0.758
**Belonging**				
Item 1	1	0	999	0.787
Item 2	0.735	0.081	*p* < .001	0.392
Item 3	0.963	0.055	*p* < .001	0.791
Item 4	0.836	0.056	*p* < .001	0.716
**Social Support**				
SS1	1	0	999	0.899
SS2	0.956	0.036	*p* < .001	0.91
SS3	1.051	0.042	*p* < .001	0.881
SS4	0.969	0.042	*p* < .001	0.858
**Regression Path**	**Estimate**	**S.E.**	***P*-value**	**R-square**
**Mistreatment predicted by**				0.403
Belonging (P1)	−0.079	0.039	0.04	
Programmatic Openness (P2)	−0.078	0.042	0.066	
Interaction (P1 X P2)	0.157	0.029	*p* < .001	
**Burnout predicted by**				0.363
Mistreatment	0.671	0.186	*p* < .001	
Belonging	−0.205	0.082	0.012	
Social Support	−0.359	0.099	*p* < .001	
Sex	−0.114	0.071	0.109	

Note: Burnout item four (WPB4) and Mistreatment item three (NAQ3) were removed in the final model due to its factor loading lower than the threshold following Hu and Bentler's [39] guidelines.

### Structural model and direct effects

The structural path of the model revealed significant protective factors of trainee mistreatment, including programmatic openness (*b* = −.23, *SE* = .10, *p* = .029), and individual belonging (*b* = −.28, S*E* = .10, *p* = .006) after controlling for demographic characteristics.

Trainee mistreatment is significantly associated with burnout (*b* = .32, *SE* = .08, *p* < .001), while social support is negatively associated with burnout (*b* = −.29, *SE* = .08, *p* < .001). Individual belonging remains a significant protective factor for burnout after controlling for social support and individual demographics, with both a direct effect (*b* = −.23, SE = .08, *p* = .008) and an indirect effect through perceived mistreatment (*b* = −.09, SE = .04, *p* = .021). The indirect effect of programmatic openness on burnout through perceived mistreatment was examined but was not statistically significant, *b* = −0.073, SE = 0.038, *p* = .056. This model has explained 40% and 36% of the total variance in trainee mistreatment and burnout, respectively.

### The moderating effects of GME programmatic openness on mistreatment

**Figure 2.**
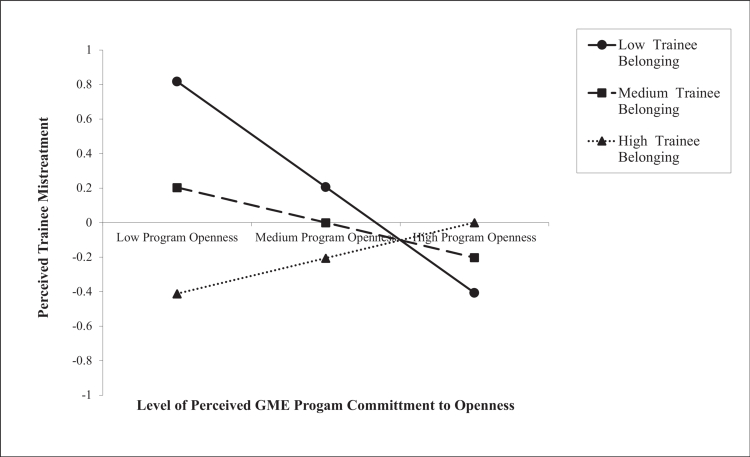
Moderation model of GME programme commitment to openness.

We proceeded with the LMS approach to examine the latent moderation effect of GME programmatic openness on trainee mistreatment (Figure 1). The results showed a significant interaction effect between programmatic openness and individual belonging on trainee mistreatment (*b* = .157, *SE* = .03, *p* < .001). As depicted in Figure 2, simple slopes showed that trainees’ reported mistreatment was the lowest when they reported a high level of individual belonging, while simultaneously perceiving their GME programme’s openness.

Simple slope analyses showed that the protective effect of GME programmatic openness was the strongest for trainees who experienced the lowest level of belonging (*b* = −.24, *SE* = .04, *p* < .001), followed by a weaker effect for trainees who experienced a medium level of belonging (*b* = −.08, *SE* = .05, *p* = .04). This effect was attenuated when trainees felt a high level of belonging (*b* = .07, *SE* = .05, *p* = .13). The attenuation is expected because the standardised overall level of misstatement was below 0 among this group (trainees with high belonging).

**Figure 3.**
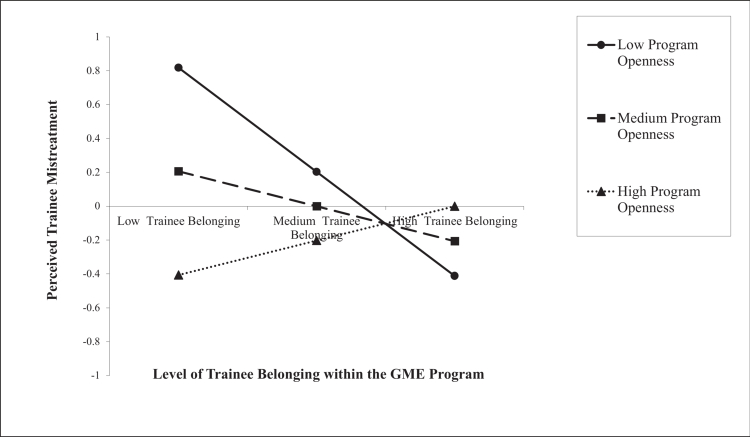
Alternative chart for the moderation model.

An alternative presentation of this interaction effect is shown in Figure 3. Perceived belonging also significantly moderated the effect of GME programmatic openness on trainee mistreatment. The protective effects of individual belonging against mistreatment peaked among trainees who experienced a low level of programmatic openness (*b* = −.24, *SE* = .05, *p* < .001). This effect is attenuated for trainees who experienced a moderate level of programmatic openness (*b* = −.08, *SE* = .04, *p* = .06) and was further attenuated for trainees who experienced a high level of programmatic openness (*b* = .08, *SE* = .05, *p* = .13).

## Discussion

Although prior research has emphasised the importance of GME programmes supporting openness and community building [42,45], much of this work has been conceptual [19,20], qualitative [46], or descriptive [17,18,32]. Existing studies on GME learning environment have generally shown that supportive and respectful climates are associated with more positive trainee experiences, yet few studies have empirically examined how programme-level climate and individual-level experiences operate together [17,32]. The current study extends this literature by testing programmatic openness as a programme-level factor associated with mistreatment and by examining how its effect varies according to trainees’ sense of belonging.

Our finding builds on prior work [17,19,20] on the importance of programme culture and aligns with ecological systems theory by showing how the local training climate interact with trainees’ day-to-day experiences of connection and acceptance [23,24]. Specifically, programmatic openness was associated with lower perceived mistreatment, but this relationship depended on trainees’ individual levels of belonging—their internal sense of acceptance, value, and connection within the training programme. From an ecological perspective, this suggests that openness and belonging operate at related but distinct levels: openness reflects a programme-level climate that may shape what can be voiced, questioned, or addressed, whereas belonging reflects how trainees experience their place within that environment. The protective effects of programmatic openness were strongest among trainees who reported low levels of belonging, suggesting that a more open training climate may partially compensate when trainees do not yet feel accepted, valued, or connected within their programme. Conversely, for GME trainees with high levels of belonging, programmatic openness had a negligible effect—likely because these individuals were already in environments where they felt valued and connected, which supported their learning and wellbeing. However, in the absence of both, trainees may be left particularly vulnerable to mistreatment and ultimately burnout.

The mechanism through which individual belonging within training programme are associated with mistreatment and burnout likely involves psychological safety [47]. Trainees who feel they belong are more likely to seek help without fear of judgement, and report concerns about inappropriate behaviour—thereby diminishing vulnerability to mistreatment and emotional exhaustion. By contrast, those who do not feel they belong may become more alert to being overlooked—such as being left out of key learning opportunities or having their input dismissed. Under these circumstances, programmatic openness may mitigate perceived mistreatment by making it safer to speak up, encouraging accepting dialogue, and modelling respectful interactions across roles.

Our finding that individual belonging was significantly associated with burnout, even after accounting for social support, is consistent with prior research showing that belonging is a distinct contributor to wellbeing rather than merely a proxy for general support [27–29]. This suggests that while a robust support system is crucial, the immediate training environment—particularly how accepted, valued, and connected trainees feel—may play an equally critical role in mitigating burnout. These findings also caution against assuming that openness automatically produces belonging, or that belonging-focused interventions eliminate the need to cultivate open programme climates. Instead, trainee wellbeing may depend on both the structural and relational conditions that allow concerns to be voiced and the day-to-day experiences that allow trainees to feel that they matter.

Prior research on ethical leadership in healthcare settings emphasises the role of open and respectful institutional climates, shaped by adaptive leaders, in supporting psychological safety and wellbeing of healthcare professionals [48]. Striking the right balance between fostering openness and maintaining professional rigour can be challenging [49], but our findings make clear that programmatic openness is not ancillary—it is foundational to trainee well-being. At the same time, openness should not be treated as synonymous with belonging. In addition to leadership commitments and policy initiatives to increase programmatic openness, efforts to improve trainee wellbeing should also attend to how trainees actually experience belonging within their everyday training environment. Understanding factors that hinder a sense of belonging—such as microaggressions, cultural mismatches, hierarchical barriers, or lack of representation [50–53]—may help inform more effective strategies. Tools such as climate surveys, reflective focus groups, or narrative-based assessments may help gauge these aspects of trainee experiences [54–56].

### Limitations and future directions

A key limitation of this study is that data were collected from a region training site with multiple GME programmes. While this approach ensured consistency in the broader institutional climate, it may limit the generalisability of findings to other institutions with different sociopolitical contexts. Furthermore, given our study is survey-based, the findings may be influenced by self-selection and nonresponse bias, as trainees with particularly positive or negative experiences may have been more likely to participate. Self-reported, cross-sectional data also limits causal inferences and may not fully capture programmatic openness in everyday training environments. Future research should explore these relationships across diverse institutional settings and examine whether similar compensatory and buffering effects emerge in other environments.

In addition, although prior literature has examined differences in climate, belonging, and mistreatment by gender, race, and other social positions [8,57–59], our sample size and composition did not provide sufficient statistical power to support reliable subgroup analyses of moderation effects [60]. As a result, we were unable to determine whether programmatic openness and belonging operated differently across trainee groups. Future studies should examine how these experiences vary based on social positioning to clarify whether some trainees remain differentially vulnerable to mistreatment and burnout. Finally, longitudinal studies could assess long-term interplays between programmatic openness and belonging on perceived mistreatment. Qualitative and mixed-methods studies are also needed to better understand how trainees interpret these experiences, and to identify concrete opportunities and strategies for strengthening an open, welcoming, and accepting climate in GME programmes that support all trainees.

### Conclusion

This study provides empirical evidence that programmatic openness is not simply a value-added feature of graduate medical education but a core component of a supportive learning environment. Our findings also show that programmatic openness and belonging, while related, are not the same construct and should not be treated as substitutes for one another. By demonstrating both additive and compensatory effects, programmatic openness emerges as a crucial buffer against mistreatment, particularly for trainees with low levels of belonging. Future efforts may consider cultivating both programmatic openness and everyday experiences of belonging through leadership practices and structural supports that attend to multiple layers of the training environment to ensure a safer learning environment for all trainees.

## Data Availability

The data supporting the findings of this study are federally owned by Uniformed Services University. Due to federal security and privacy restrictions, the datasets are not publicly available. Data are, however, available from the corresponding author upon reasonable request and with the permission of Uniformed Services University.
